# Emulsion-Templated Porous Polymers for Efficient Dye Removal

**DOI:** 10.1021/acsomega.2c01472

**Published:** 2022-04-29

**Authors:** Gülenay Üzüm, Büşra Akın Özmen, Ebru Tekneci Akgül, Erdem Yavuz

**Affiliations:** Department of Chemistry, Istanbul Technical University, Maslak-Istanbul 34469, Turkey

## Abstract

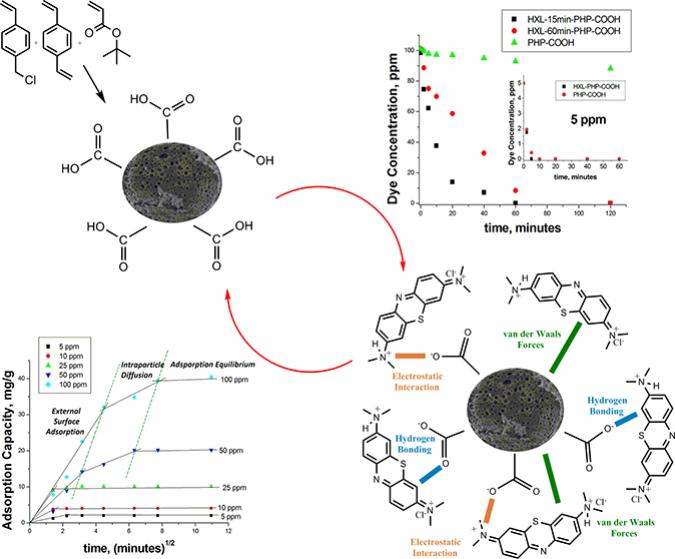

A high internal phase emulsion (HIPE) method was used to produce adsorbents with an interconnected porous structure. HIPE was prepared using vinyl benzyl chloride (VBC), divinylbenzene (DVB), *tert*-butyl acrylate, and Span80 as the organic phase and water with K_2_S_2_O_8_ and CaCl_2_ as the water phase. The polymerization of the organic phase produced highly porous polymers called polyHIPE, carrying two functional groups. As a result of the template method, polyHIPEs have a low surface area. To overcome this drawback, polyHIPE was hyper-cross-linked through VBC to create meso- and micropores, resulting in a higher surface area. Then the polymer surface was tailored with carboxylic acid groups by simple hydrolysis of *tert*-butyl acrylate. The adsorption performances of the acidic functional hyper-cross-linked polyHIPEs prepared for the various reaction times of 0, 15, and 60 min were compared for methylene blue. The hyper-cross-linked polyHIPEs showed an enhanced adsorption kinetics for methylene blue, and the 15 min hyper-cross-linking reaction increased the rate of methylene blue adsorption significantly. It was proven that the polyHIPE adsorbent can be reused by treating it with an aqueous acidic solution in ethanol.

## 1 Introduction

Various hazardous substances released from industrial and municipal wastes cause serious environmental problems. In particular, synthetic dyes are very persistent in nature and cause harmful effects on aquatic ecosystems as well as human health.^1,2^ Tons of dyes are directly discharged into global water systems from textile industries annually.^3^ These dyes in water can be easily distinguishable even in trace amounts. Some dyes are detectable even at concentrations less than 1 ppm, coloring a large volume of water bodies. The penetration of sunlight is blocked by dye molecules from the water surface, reducing photosynthesis and inhibiting plant growth.^4,5^ Methylene blue (MB) is a widely used cationic dye having small molecular size that is used as an antiseptic and for other medicinal purposes, for dyeing, printing cotton and tannin, indicating oxidation–reduction.^6,7^ Although MB is not strongly hazardous, it has various harmful effects. Toxicity of methylene blue was found on two microalgae, viz. *Chlorella vulgaris* and *Spirulina platensis*.^8^ Microalgae are ecologically important species, and the inhibition of growth of microalgae due to dye pollution causes severe environmental damage. Moreover, MB is a potential carcinogen because of the aromatic amines.^9^ Therefore, it is necessary to remove methylene blue from aqueous solutions. A number of different technologies^10−12^ have been developed to recover and reduce dyes from wastewater, such as photocatalytic degradation of dye contaminants with TiO_2_ immobilized on Zsm-5 zeolite modified with nickel nanoparticles^13^ and efficient degradation and detoxification of methylene blue dye by a newly isolated ligninolytic enzyme producing bacterium *Bacillus albus* Mw407057,^14^ silsesquioxane-based hybrid materials.^15,16^ One approach has been the use of porous polymers with various functional groups.^17^ In this way, efficient dye removal is possible without producing secondary pollution.^18,19^ Polymeric adsorbents have been widely used to remove dyes very effectively and even selectively through physical dye removal. They have advantages over other methods such as simplicity, easy preparation and scale-up, recyclability, easy functionalization, as well as wide range of available monomers to prepare them. They can be prepared to be highly porous with high surface areas and very good candidates for removal of dyes from wastewater as efficient adsorbents. Various polymeric adsorbents have been prepared for a successful adsorption of methylene blue such as polydopamine microsphere-incorporated electrospun fibers,^20^ agar/κ-carrageenan composite hydrogel adsorbents,^21^ H_2_SO_4_ cross-linked magnetic chitosan nanocomposite beads,^22^ a poly(acrylic acid) (PAA)-based superadsorbent nanocomposite hydrogel (NC gel),^23^ and functional porous organic polymers with conjugated triaryl triazine.^24^

High internal phase emulsion (HIPE) methods are templates with the internal phase volume ratio of 0.74 or greater. The internal phase is water in the case of a water-in-oil emulsion, and curing the external or monomer phase forms highly porous polymers named polyHIPEs.^25−30^ PolyHIPEs have hierarchical and interconnected porosity with some properties such as easy preparation, tunable porosity, and open cellular morphology. By removing the droplet phase (generally water), large voids are formed, and by tuning the surfactant ratio, interconnections between voids are created as a result of thinning of the polymer walls around the droplets.^25^ These highly porous materials possess primarily macropores, resulting a low surface area in a range between 3 and 10 m^2^/g. In the adsorption process, low surface area is a huge disadvantage, causing slow adsorption kinetics. However, the polyHIPE surface can be easily enhanced through hyper-cross-linking reaction using Friedel–Crafts alkylation.^31,32^

In this work, the polymeric adsorbents with carboxylic acid groups were prepared using an emulsion-templated method. First, a high internal phase emulsion was prepared using vinyl benzyl chloride (VBC), divinylbenzene (DVB), *tert*-butyl acrylate, and Span80 as the organic phase and water with K_2_S_2_O_8_ and CaCl_2_ as the water phase. Here, *tert*-butyl acrylate as the monomer was chosen to tailor the polymer surface with acidic functions. Due to the fact that esters can be easily hydrolyzed in strong acidic and basic solutions, the carboxylic acid groups were obtained on the polymer surface via hydrolysis. DVB is a cross-linker, and it was used at 10% (mol %) regarding the total monomer phase. The monomer phase also contains VBC to introduce the alkyl chloride groups onto the polymer surface. PolyHIPEs were hyper-cross-linked through alkyl chloride groups to increase the surface area. Both the acidic functional hyper-cross-linked and unhyper-cross-linked counterparts were compared for MB adsorption to observe the advantage of the hyper-cross-linking reaction.

## 2 Materials and Methods

### 2.1 Materials

Vinyl benzyl chloride (Fluka), divinylbenzene (Fluka), styrene (Fluka), and *tert*-butyl acrylate (Sigma-Aldrich) were passed through an alumina column to remove the inhibitors. FeCl_3_ (anhydrous, Merck), calcium chloride dihydrate (CaCl_2_·2H_2_O, Sigma-Aldrich), 1,2-dichloromethane (Lab-Scan Analytical Sciences), potassium persulfate (K_2_S_2_O_8_, Sigma-Aldrich), nitric acid (Merck), ethanol (95%, Carlo Elba), isopropyl alcohol (IPA, technical grade), and acetone (technical grade) were used as received.

### 2.2 PolyHIPE (90% Pore Volume) Preparation

Initially, emulsion-templated polymers (polyHIPE) were prepared and hyper-cross-linked using FeCl_3_ as the catalyst followed by the hydrolysis reaction to produced porous carboxylic acid functional polymeric adsorbents.

To prepare a stable HIPE, a 250 mL round-bottomed flask was charged with the monomer phase consisting of VBC (4 mL), *tert*-butyl acrylate (4 mL), DVB (2 mL), and surfactant Span80 (200 mg). An overhead stirrer with a D-shaped paddle with the diameter of 6 cm was connected to this flask containing the monomer phase, with a total volume of 10 mL, and purged with nitrogen for 15 min. Then in a separate flask, potassium persulfate (200 mg) as the initiator and calcium chloride dihydrate (1000 mg) as the electrolyte were dissolved in 90 mL of distilled water as the internal phase and transferred to a dropping funnel connected to the flask containing the monomer phase. Next, the internal phase was purged with nitrogen for 15 min. At the time, the nitrogen supply was removed, and the internal phase was added to the flask with stirring at 350 rpm drop by drop for 30 min. The mechanical stirring continued for an additional 1 h at 350 rpm to form a homogeneous emulsion. At the end of the process, a very viscous stable emulsion (HIPE) was obtained. A 50 mL PET centrifuge tube as a mold was charged with the prepared HIPE and cured at 60 °C for 48 h. To remove the surfactant and polymerization impurities, polyHIPE in the shape of monolith was washed in a Soxhlet extractor with distilled water and isopropyl alcohol, both for 24 h, and then dried in a conventional oven at 60 °C for 24 h.

### 2.3 Hyper-Cross-Linking Reaction of PolyHIPE (HXL-PHP)

To obtain high surface area polymers, polyHIPE (PHP) was hyper-cross-linked. For this purpose, initially the monolith polyHIPE was powdered using a mortar and pestle. A 250 mL three-neck round-bottom flask connected with a reflux condenser was charged with DCE (50 mL) and PHP (1000 mg) and then purged with nitrogen through a rubber septum and needle for 15 min. The flask was stirred for 1 h at 300 rpm at room temperature to swell the polymer and placed in an ice bath. Next, anhydrous FeCl_3_ (1000 mg) was added to the flask quickly and purged with nitrogen again for 15 min. To make sure a uniform dispersion of FeCl_3_ was obtained, the flask was left stirring for an additional 30 min and then it was brought to room temperature. The swollen polyHIPE was placed in an oil bath to perform the Friedel–Crafts alkylation reaction for 15 min. At the end of the reaction, ethanol (50 mL) was poured into the flask to stop the reaction. The reaction mixture was filtered under vacuum and washed with ethanol (3 × 30 mL) and 0.1 M HNO_3(aq)_ (3 × 30 mL), and then the hyper-cross-linked polyHIPE was placed in a Soxhlet extractor, washed with acetone for 8 h (12 cycles), and dried in a conventional oven at 60 °C. This hyper-cross-linking reaction procedure was repeated for 60 min.

### 2.4 Hydrolysis of PolyHIPE and HXL-PHP

PHP (1000 mg) was placed in a 250 mL round-bottom flask together with dioxane (10 mL) as solvent and HCl (4 mL) at room temperature. This mixture was refluxed for 6 h and then cooled to room temperature. The hydrolyzed polyHIPE (PHP-COOH) was put in a beaker containing dioxane (40 mL), and then it was filtered under vacuum and washed sequentially with ethanol (2 × 20 mL), distilled water (3 × 30 mL), and ethanol (20 mL). PHP-COOH was dried under vacuum at 40 °C for 24 h. This procedure was repeated to hydrolyze *tert*-BuA functional HXL-PHPs (prepared with 15 and 60 min hyper-cross-linking reactions: HXL-15 min-PHP-COOH and HXL-60 min-PHP-COOH, respectively).

### 2.5 Functional Group Content Determination

The carboxylic acid contents of PHP-COOH, HXL-15 min-PHP-COOH, and HXL-60 min-PHP-COOH were determined by acid–base back-titration. For this purpose, the polymer sample (25 mg) was placed in a flask charged with 0.1 M NaOH_(aq)_ and stirred for 24 h at room temperature. Then this mixture was filtered under gravity, and 1 mL of the filtrate was transferred into a 10 mL flask. The filtrate was titrated with 0.01 M HCl solution in the presence of a phenolphthalein indicator to determine the acid group capacity of the porous polymers.

### 2.6 MB Batch Adsorption/Desorption Experiments

#### 2.6.1 Determination of the Optimal Adsorbent Amount

To obtain the optimal amount of adsorbent, the acidic functional polyHIPEs (10–100 mg) were contacted with a 10 mL dye solution (100 ppm) and stirred for 3 h at 400 rpm in the centrifuge bottles using a magnetic stir bar for 3 h. Then each bottle was centrifuged at 5000 rpm for 5 min to separate the solution phases. The amount of methylene blue adsorbed onto polyHIPE adsorbents was found by the absorbance at 663 nm after and before adsorption using a UV–vis spectrophotometer.

#### 2.6.2 Effect of pH on Methylene Blue Adsorption

The polymeric adsorbents (25 mg, optimal amount) were placed in a 15 mL conical bottom centrifuge tube with 10 mL of aqueous methylene blue solution (100 ppm) to find the dye sorption capacities of the adsorbents. The dye solutions with various pH values (4–8) were mixed with the polymer samples and stirred at 400 rpm in the centrifuge bottles using a magnetic stir bar for 3 h. The amount of methylene blue adsorbed onto polyHIPE adsorbents was found, as explained in section 2.6.1.

#### 2.6.3 Effect of Ionic Strength on Methylene Blue Adsorption

To study the effect of ionic strength of the adsorption medium on the dye–polymer interaction, NaCl_(aq)_ solutions with various salt concentrations from 0.1 to 1.0 M were used. The optimal amount of the adsorbent (25 mg) was placed in a 15 mL conical bottom centrifuge tube with 10 mL of aqueous methylene blue solution (100 ppm dye concentration, pH 7, and 0.1–1.0 M salt concentration). These polymer–dye solution mixtures were stirred at 400 rpm in the centrifuge bottles using a magnetic stir bar for 3 h. The amount of methylene blue adsorbed onto polyHIPE adsorbents was found, as explained in section 2.6.1.

#### 2.6.4 Determination of the Maximum Adsorption Capacity of the Adsorbents

To find the maximum amount of methylene blue adsorbed on the polymeric adsorbents, dye adsorption experiments were carried out with various methylene blue concentrations from 5 to 1500 ppm. The adsorption experiments were performed as explained before, and the results were used to fit three adsorption isotherms, namely, Langmuir, Freundlich, and Dubinin–Radushkevich (D–R).

#### 2.6.5 Dye Adsorption Kinetics of the Adsorbents

To investigate the optimal hyper-cross-linking degree for methylene blue adsorption on polyHIPE adsorbents, batch adsorption kinetic experiments were performed. The adsorption kinetics results of PHP-COOH, HXL-15 min-PHP-COOH, and HXL-60 min-PHP-COOH were compared.

#### 2.6.6 Investigation of the Selectivity of HXL-15 min-PHP-COOH

To investigate the adsorption selectivity of HXL-15 min-PHP-COOH toward methylene blue, batch adsorption experiments were carried out with an acidic dye, namely, reactive red, and a basic dye, namely, malachite green, as described before. The maximum dye adsorption capacities and the kinetic performances were compared to observe the selectivity of the adsorbent.

#### 2.6.7 Reuse of the Polymeric Adsorbent

The desorption capacity and recyclability of HXL-15 min-PHP-COOH was studied using an acidic solution. The polymeric adsorbent (25 mg) loaded with methylene blue was placed in a 15 mL conical bottom centrifuge tube with 10 mL of 1 M HCl/ethanol solution (50%, v/v). This mixture was stirred at 400 rpm in a centrifuge bottle using a magnetic stir bar for 3 h and then centrifuged at 5000 rpm for 5 min to separate the solid and solution phases. The amount of methylene blue released from the adsorbent surface was found by the absorbance after and before adsorption using a UV–vis spectrophotometer. The desorbed polymeric adsorbent was washed with distilled water three times before the adsorbent was charged with methylene blue to remove any nonbonded dye molecules from the polymer surface.

#### 2.6.8 Polymer Characterization

The porous polymers were characterized by various methods: scanning electron microscopy (SEM, FEI-Philips XL30 Environmental scanning electron microscope with a field emission gun equipped with an energy-dispersive X-ray analysis unit) operating at 10.0 kV was used to observe the surface morphology. The samples were prepared by dispersing the powder onto a double-sided adhesive surface. The qualitative determination of the surface functional groups was carried out by Fourier transform infrared (FTIR) spectroscopy (Thermo Scientific, Nicolet iS20) in the range between 4000 and 450 cm^–1^. The surface elemental analysis was identified by X-ray photoelectron spectroscopy (XPS) measurements (Thermo Scientific K-Alpha X-ray photoelectron spectrometer) in the range between 100 and 4000 eV. The nitrogen adsorption isotherms were measured at −196 °C using a Micromeritics TriStar II 3020 surface area and pore size analyzer. The samples were degassed for 12 h at 100 °C before the measurements. The average pore size was determined using the Barrett–Joyner–Halenda (BJH) method. Dye concentrations in the adsorption experiments were determined by a double beam UV–vis spectrophotometer (PerkinElmer, Lambda 25).

## 3 Results and Discussion

In this work, the polymeric adsorbents with carboxylic acid groups were prepared with an emulsion templated method. First, a high internal phase emulsion was prepared using vinyl benzyl chloride, divinylbenzene, *tert*-butyl acrylate, and Span80 as the organic phase and water with K_2_S_2_O_8_ and CaCl_2_ as the water phase. Here, *tert*-butyl acrylate as the monomer was chosen to tailor the polymer surface with acidic functions. To obtain acidic groups on the polymer surface, acrylic acid or methacrylic acid monomers could be used; however, these monomers are hydrophilic. Hydrophilic monomers do not remain in the organic (monomer) phase and can destabilize the emulsion. A hydrophilic polyHIPE can be prepared using an oil-in-water (O/W) HIPE;^33−35^ however, O/W HIPEs have poor stability, resulting in polyHIPEs with low porosities.^36^ Therefore, *tert*-butyl acrylate was used in the monomer phase to obtain a stable HIPE. It is a fact that esters can be easily hydrolyzed in strong acidic and basic solutions. Carboxylic acid groups were obtained on the polymer surface via hydrolysis. Divinylbenzene is a cross-linker, and it was used as 10% (mol %) of the total monomer phase. It is necessary to use a cross-linker to obtain a network structure, and also, it enhances the emulsion stability. The monomer phase also contains vinyl benzyl chloride to introduce the benzyl chloride groups onto the polymer surface. Because polyHIPEs possess mainly macropores (pores larger than 50 nm), they have low surface areas (3–10 m^2^/g). To enhance the surface area, polyHIPEs were hyper-cross-linked through a Friedel–Crafts alkylation reaction catalyzed by a Lewis acid, FeCl_3_.

### 3.1 Preparation of High Internal Phase Emulsion and PolyHIPE

In the HIPE formation, the continuous phase (monomer phase) contains vinyl benzyl chloride, divinylbenzene, *tert*-butyl acrylate, and surfactant Span80, and the water phase is composed of distilled water, initiator K_2_S_2_O_8_, and electrolyte CaCl_2_. The water phase was added to the organic phase slowly, and with polymerization of the monomer phase, a low density and permeable, highly porous material, namely, polyHIPE, was obtained. The surfactant Span80 stabilizes the emulsion, and it is necessary to obtain an open-porous, interconnected network. During the curing, polymer walls in droplets become thinner, and eventually pores form between droplets (windows). Therefore, it is very crucial to use a proper amount of surfactant to make sure that the resulting polymer has an interconnected porous structure. After the droplet phase was removed by drying, the large pores (voids) and the interconnecting pores are formed.

### 3.2 Synthesis of Polymeric Adsorbents

**Scheme 1:**
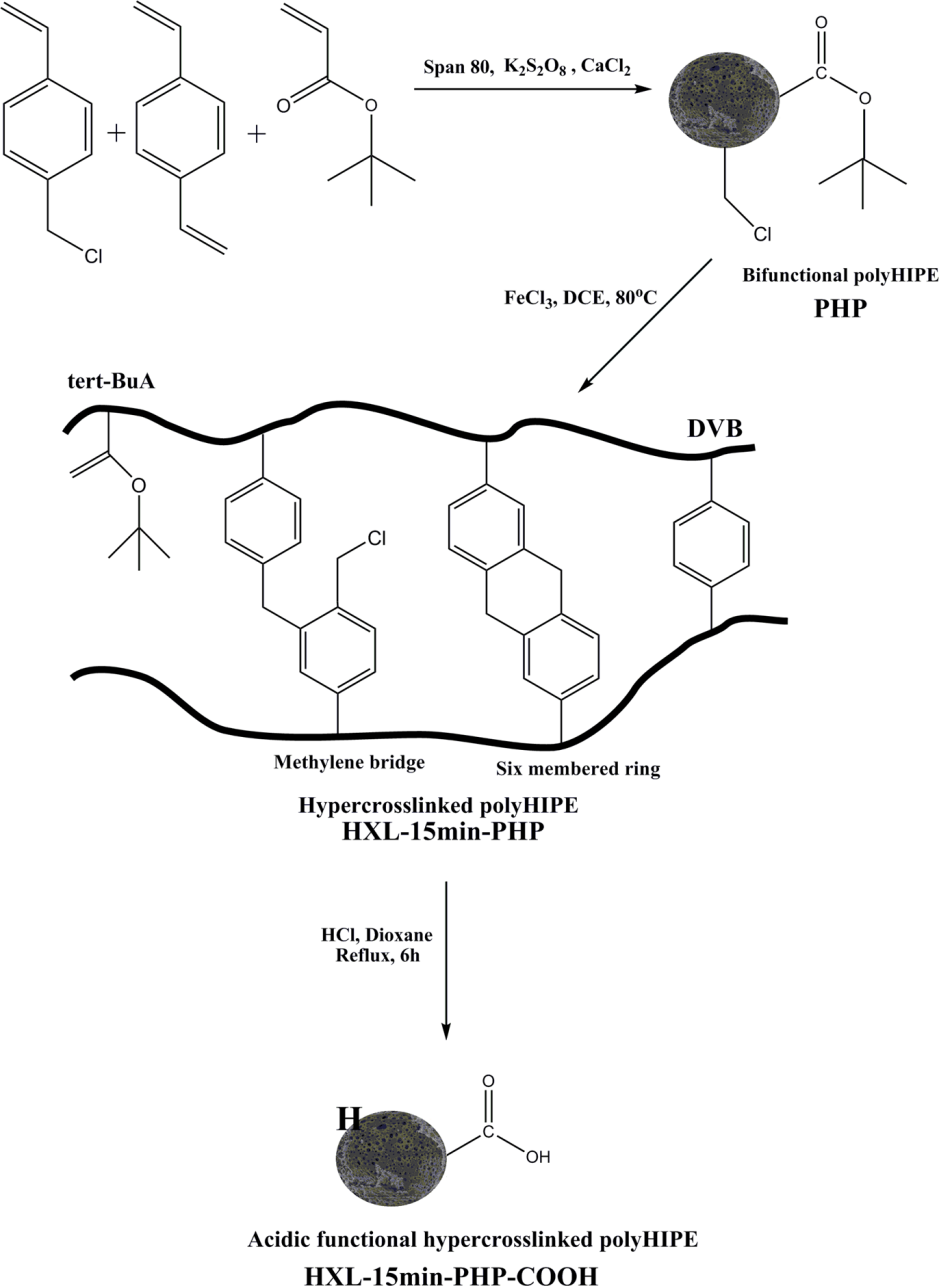
Preparation of the Carboxylic Acid Functional PolyHIPE and the Probable Cross-Linking Formations during the Hyper-Cross-Linking Reaction

A three-step procedure is used to prepare an acidic functional polymeric adsorbent: (1) a bifunctional polyHIPE containing *tert*-BuA and vinyl benzyl chloride as a polymer support; (2) the hyper-cross-linking reaction through alkyl chloride groups to increase the surface area of polyHIPE; (3) the hydrolysis of HXL-PHP to introduce the carboxylic acid groups to the polymer surface (Scheme 1). With this procedure, the polyHIPE surface could be tailored with acidic groups covalently bonded, and the adsorbent can bind basic methylene blue dye.

In addition, *tert*-butyl acrylate, DVB, and VBC were used to prepare a polyHIPE, followed by the hydrolysis reaction carried out in dioxane catalyzed by hydrochloric acid. This carboxylic acid functional polymeric adsorbent was used to compare the adsorption kinetics performance of the hyper-cross-linked polymer for dye adsorption. To obtain a polymer with high surface area, the Friedel–Crafts alkylation reaction catalyzed by a Lewis acid, FeCl_3_, was carried out, with the chloromethyl groups acting as internal electrophiles and dichloroethane (DCE) as solvent. DCE has a boiling point of 80 °C, so this solvent both allows the reaction to take place at high temperatures and also acts as an external cross-linker for the hyper-cross-linking reaction. The surface area of the polyHIPE precursor was increased by micro- and mesopores created through the formation of methylene bridges and six-membered rings between the monomer units of VBC. Also, DCE as an external cross-linker may be involved in the bridge formation, as well. The *tert*-BuA groups remain on the polymer surface after the hyper-cross-linking reaction, so further hydrolysis gives an acid functional hyper-cross-linked porous polymer (Scheme 1).

### 3.3 Physicochemical Properties of the Adsorbents

**Figure 1:**
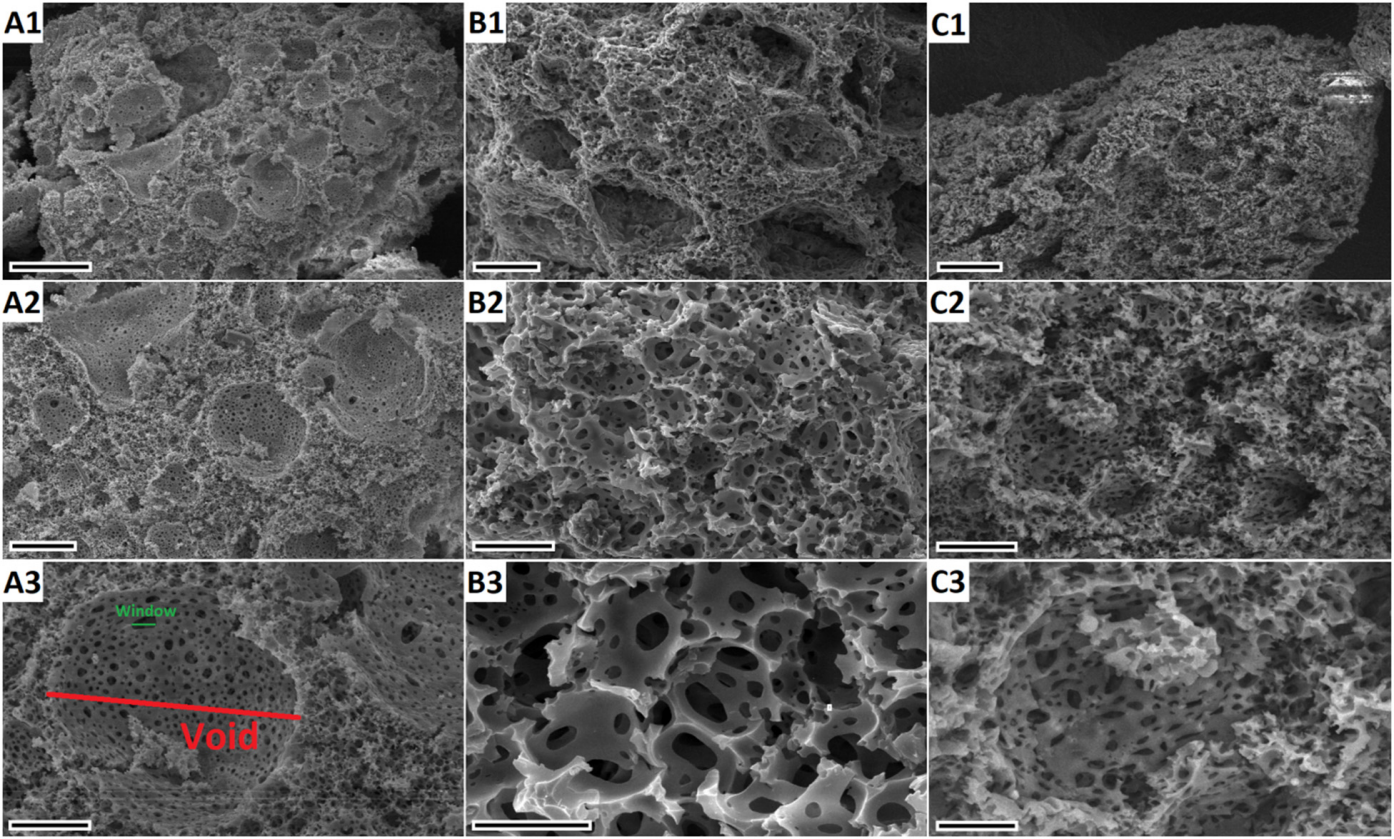
(A1–A3) SEM images of polyHIPE (PHP), (B1–B3) HXL-15 min-PHP, and (C1–C3) HXL-15 min-PHP-COOH. Scale bars: (A1) 50 μm; (A2) 20 μm; (A3) 10 μm; (B1,C1) 20 μm; (B2,C2) 10 μm; (B3,C3) 5 μm.

To characterize the surface morphology of the porous polymers, SEM was used. PolyHIPE morphology is complex, so the terminology created by Cameron and Barbetta was preferably used. They defined the large spherical pores as voids and the smaller pores which interconnect voids as windows.^30^ In Figure 1, the hierarchical pore structure of polyHIPE material can be clearly seen. It shows a typical polyHIPE morphology which contains macropores, voids, and windows. In this study, the porous polymers were prepared with 90% porosity, and voids with a distribution between 5 and 20 μm were observed based on the SEM images. As can be observed from the SEM images, the polymer surface has plenty of windows interconnecting voids. The size of the windows are have a diameter of 1–2 μm based on the SEM images. The functional groups attached to the interior of the voids bind the dye molecules. One of the main problems of producing polyHIPEs is the destabilization of the emulsion during HIPE formation. High internal phase emulsions are destabilized with time, so HIPE stabilization must be kept until the polymerization is completed. Otherwise, the cellular structure of polyHIPE with interconnectivity cannot be obtained. Therefore, to prove that the prepared polyHIPEs have the same morphology all over the framework, the SEM images at various scale bars were taken. As can be seen from Figure 1, the characteristic morphology of the polyHIPE samples was sustained all over the frameworks for all three samples.

Figure 1 shows the SEM images of the HXL-15 min-PHP (B1–B3). As can be seen, the hyper-cross-linked polyHIPE also has a cellular formation. The hyper-cross-linking reaction creates micro- and mesopores through methylene bridges and six-membered rings. In this way, the surface area of the porous polymer can be increased without changing the surface morphology significantly, and it can be possible to produce a polymer with a higher surface area and interconnected porosity, allowing all surface active groups to be available. Also, to observe the effect of hydrolysis on polyHIPE morphology, the SEM images of the hydrolyzed HXL-PHP were also taken. As can be seen in Figure 1, the cellular morphology of polyHIPE remained similar after the hydrolysis reaction (C1–C3). The larger voids formed during the vaporization of the droplet phase, and the windows formed as a result of thinning of the polymer walls upon curing. This result proves that the porous polymer can be used as an adsorbent with the interconnected, open-porous structure. Moreover, two main pores in the polyHIPE structure can be seen in Figure 1A3, with voids and windows. Due to the large and interconnected open pores, polyHIPEs allow adsorbates on the surface active groups by mass transfer over diffusion. This is the main advantage of polyHIPE-based adsorbents. As can be seen from Scheme 1, the hyper-cross-linking reaction introduces additional cross-links together with DVB. It is a controlled reaction, so the surface area of the polymer can be increased in a controlled manner, and further functionalization through hydrolysis forms a carboxylic acid functional hyper-cross-linked polymer. To study the hyper-cross-linking effect on methylene blue adsorption, we prepared 15 min- and 60 min-HXL-PHPs, and it was observed that the HXL-15 min-PHP-COOH showed very fast adsorption kinetics, and a further hyper-cross-linking reaction did not enhance the adsorption performance of polyHIPE adsorbents.

**Figure 2:**
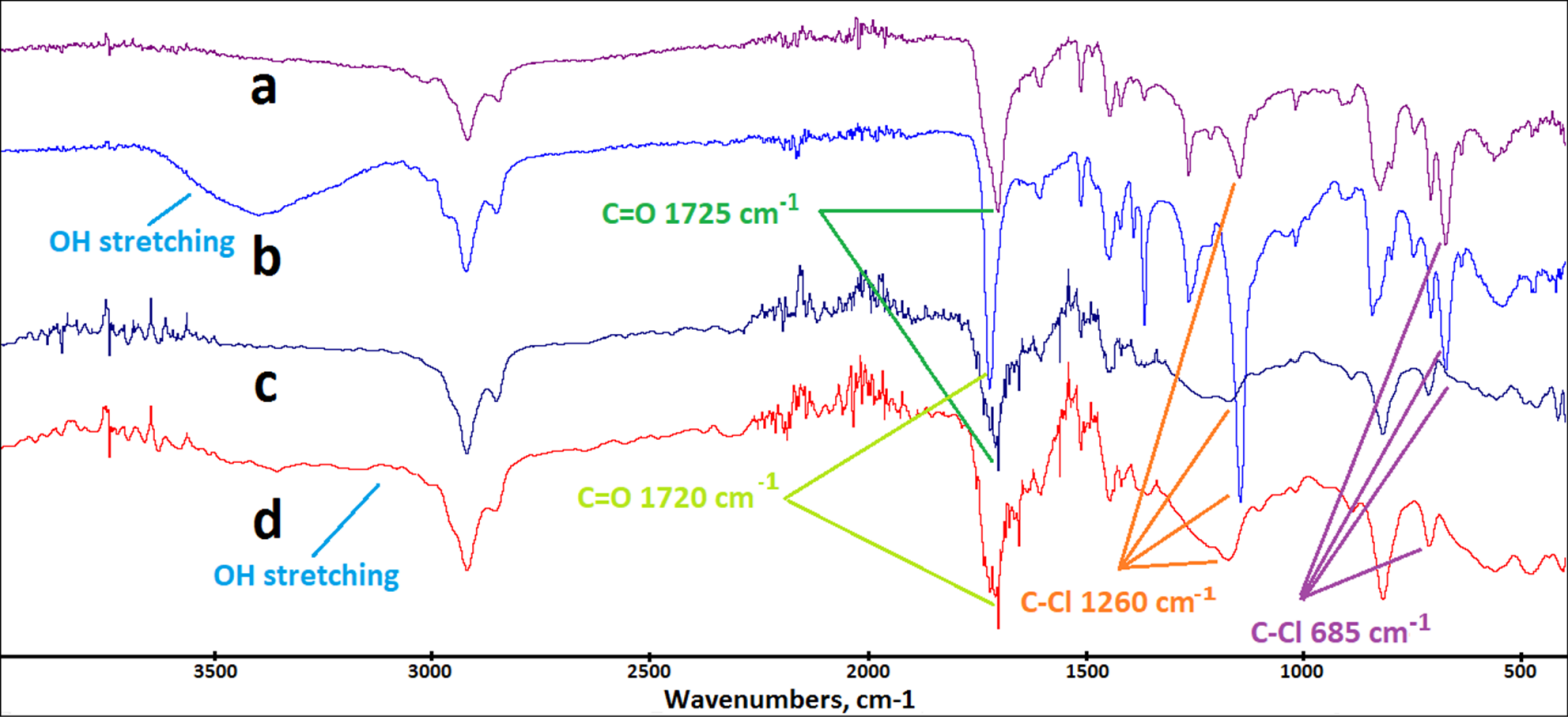
FTIR spectra of the polymers: (a) PHP, (b) PHP-COOH, (c) HXL-15 min-PHP, and (d) HXL-15 min-PHP-COOH.

The surface functional groups were determined by FTIR analysis. In Figure 2, the FTIR spectra of the polymers (PHP, PHP-COOH, HXL-15 min-PHP, and HXL-15 min-PHP-COOH) can be observed. The characteristics of the aromatic ring arise from the polyHIPE, as proven by the peak observed around 1480 cm^–1^ for all four polymers (Figure 2). It can also be observed that a strong peak at 1725 cm^–1^ (PHP, Figure 2a, and HXL-15 min-PHP, Figure 2c) originates from the carbonyl (C=O) stretching vibrations. This ester carbonyl peak shifts from 1725 to 1720 cm^–1^ may be attributed to the H-bonding with the −OH group of −COOH, which was produced due to hydrolysis. Moreover, we observed broad OH stretching vibrations between 3000 and 3600 cm^–1^ (Figure 2b,d) as clear evidence of a successful hydrolysis reaction. Also, FTIR can confirm the hyper-cross-linking reaction: C–Cl stretching vibration (685 cm^–1^ and around 1200 cm^–1^) peaks originating from vinyl benzyl chloride pendant groups were significantly diminished after the Friedel–Crafts reaction. Therefore, it can be concluded by the spectroscopy data that the hydrolysis and hyper-cross-linking reactions were performed successfully to produce the targeted polymers.

**Figure 3:**
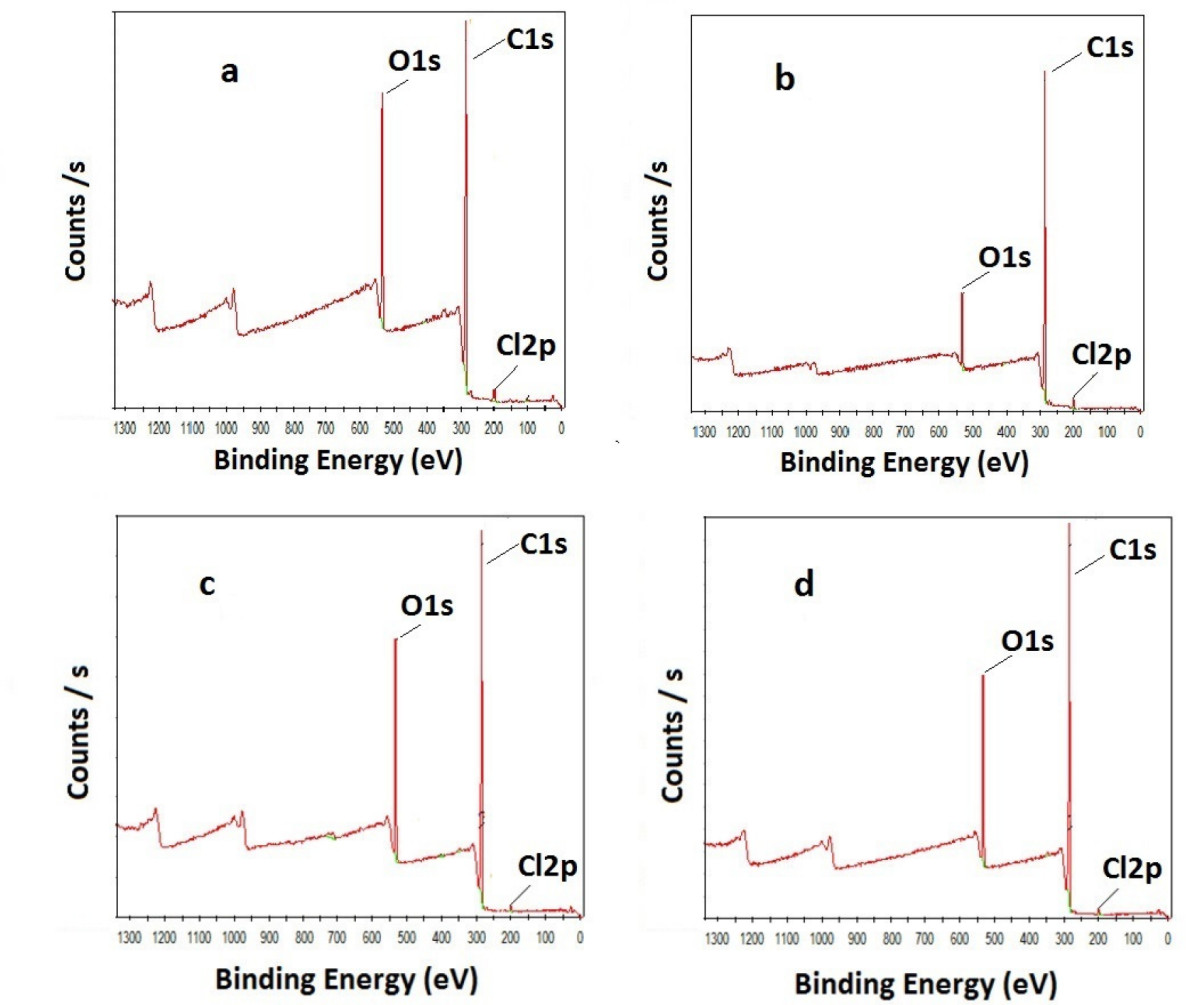
XPS surveys of (a) PHP-COOH, (b) PHP, (c) HXL-15 min-PHP-COOH, and (d) HXL-15 min-PHP.

The surface elemental analysis of polyHIPE materials was confirmed by both XPS and acid–base back-titration. With the surface elemental analysis, the functional group content of the polymeric adsorbents can be found. In Figure 3, XPS surveys of PHP-COOH (Figure 3a) and PHP (Figure 3b) can be seen. For both polymers, the expected element peaks, which are C, O, and Cl, were observed. Moreover, the elemental compositions and functional group capacities were found from the peak areas in the surveys. In agreement with the calculations, 10.49% O content corresponds to a 3.28 mmol/g total functional group capacity for the polyHIPE, and 18.77% O indicates a 5.82 mmol/g total functional group for PHP-COOH. As can be seen in Table 1, the functional group capacities of the polymers calculated from XPS and titration are close to each other. The slightly lower capacity compared to that of the theoretical one might come from the fact that some *tert*-BuA monomers were not involved in polymerization. Due to the tertiary butyl group, which leaves via hydrolysis, the functional group amount per gram of polymer increases, so PHP-COOH has a functional group content higher than that of PHP. The functional group capacities of HXL-15 min-PHP and HXL-15 min-PHP-COOH calculated from XPS and titration are also in good agreement. The high functional group capacity based on XPS results for HXL-15 min-PHP-COOH (20.49% O, Figure 3c) and HXL-15 min-PHP (18.66% O, Figure 3d) could be the adsorption of oxygen on the higher surface area polymers during XPS measurements. We can also observe Cl 2p peaks in all surveys in Figure 3, which can be explained by the short hyper-cross-linking reaction time (15 min). As a result of this short period of time, some VBC groups might not be involved in the hyper-cross-linking reaction.

**Table 1:** Functional Group Analysis

functional group content (mmol/g)				
	PHP	PHP-COOH	HXL-15 min-PHP	HXL-15 min-PHP-COOH
XPS	3.28	5.62	5.39	5.8
titration		4.80		4.9
theoretical^a^	3.61	4.53^b^		

a Calculated based on the initial monomer amounts.

b By leaving the tertiary butyl group, the functional group amount per gram of polymer increases.

**Figure 4:**
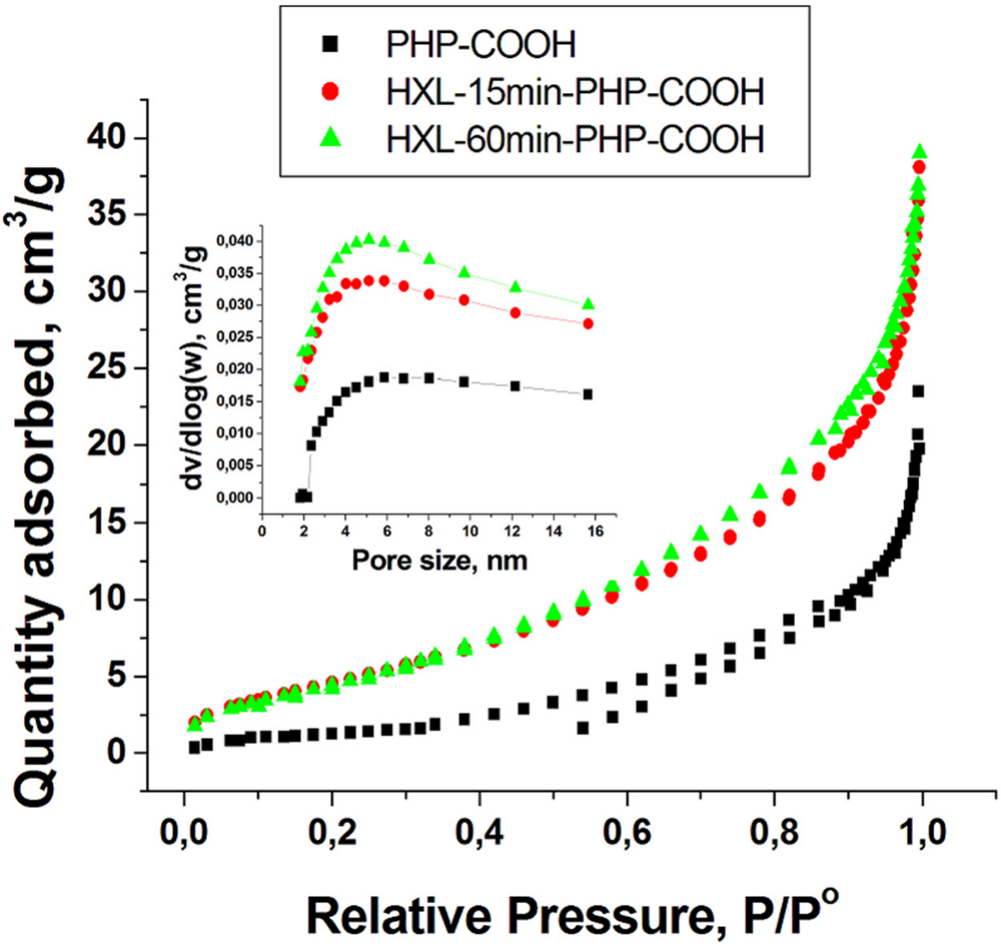
Nitrogen adsorption–desorption isotherms for the adsorbents. Inset shows the pore size distributions.

Hyper-cross-linked polymers are described by various pore size distributions and specific surface areas. The hyper-cross-linking reaction creates micro- and mesopores in macroporous the polyHIPE framework, resulting in an increase in the surface area. The surface area and porosity of the materials were analyzed by the Brunauer–Emmett–Teller (BET) isotherm model. Based on the N_2_ adsorption isotherms given in Figure 4, the BET surface area (*S*_BET_), pore volume (*V*_p_), and average pore size are obtained and given in Table 2. We can observe from the data presented (Table 2) that the lowest surface area (*S*_BET_ = 4.50 m^2^/g) was observed for the unhyper-cross-linked acidic functional polyHIPE due to its macroporous structure that possesses large voids and windows. However, the 15 min hyper-cross-linking reaction caused a 4-fold increase in the surface area (*S*_BET_ = 18.70 m^2^/g) of the PHP precursor. We also wanted to see if the further reaction could increase the surface area. As can be seen in Table 2, the polymer surface remained almost the same after the 60 min hyper-cross-linking reaction. Therefore, we chose HXL-15 min-PHP-COOH for the dye adsorption experiments. It was concluded that the reason why the surface area does not increase by the higher extension of the hyper-cross-linking reaction could be the fact that PHP precursors were prepared with a 10% DVB ratio. This can cause poor swelling and limits the surface area of the resulting hyper-cross-linked polymer. The attempts to prepare polyHIPE with the lower DVB ratio failed because of the shrinkage of polyHIPE monolith upon drying. Also, the VBC/*tert*-BuA ratio was kept not so high to produce more acidic functions. The low amount of VBC can cause a low surface area. Although the hyper-cross-linked polymers in this study do not have very high surface area in comparison with the hyper-cross-linked polymers reported in literature,^31^ HXL-15 min-PHP-COOH showed very fast kinetics compared to those of the unhyper-cross-linked counterpart. Furthermore, an increase in mesoporosity confirmed by the pore size distribution based on the BJH method as a result of the hyper-cross-linking reaction can be observed (Figure 4, inset). The surface area and porosity analysis for all of the adsorbents can be found in Table 2.

**Table 2:** Surface Area and Porosity Analysis of the Adsorbents

	from BET measurements		
sample ID	*S*_BET_ (m^2^/g)	*V*_p_^a^ (cm^3^/g)	average pore size^b^ (nm)
PHP-COOH	4.50	0.015	5.78
HXL-15 min-PHP-COOH	18.70	0.029	4.95
HXL-60 min-PHP-COOH	18.96	0.033	4.95

a *V*_p_: pore volume.

b Based on BJH adsorption.

### 3.4 Dye Adsorption Results

**Figure 5:**
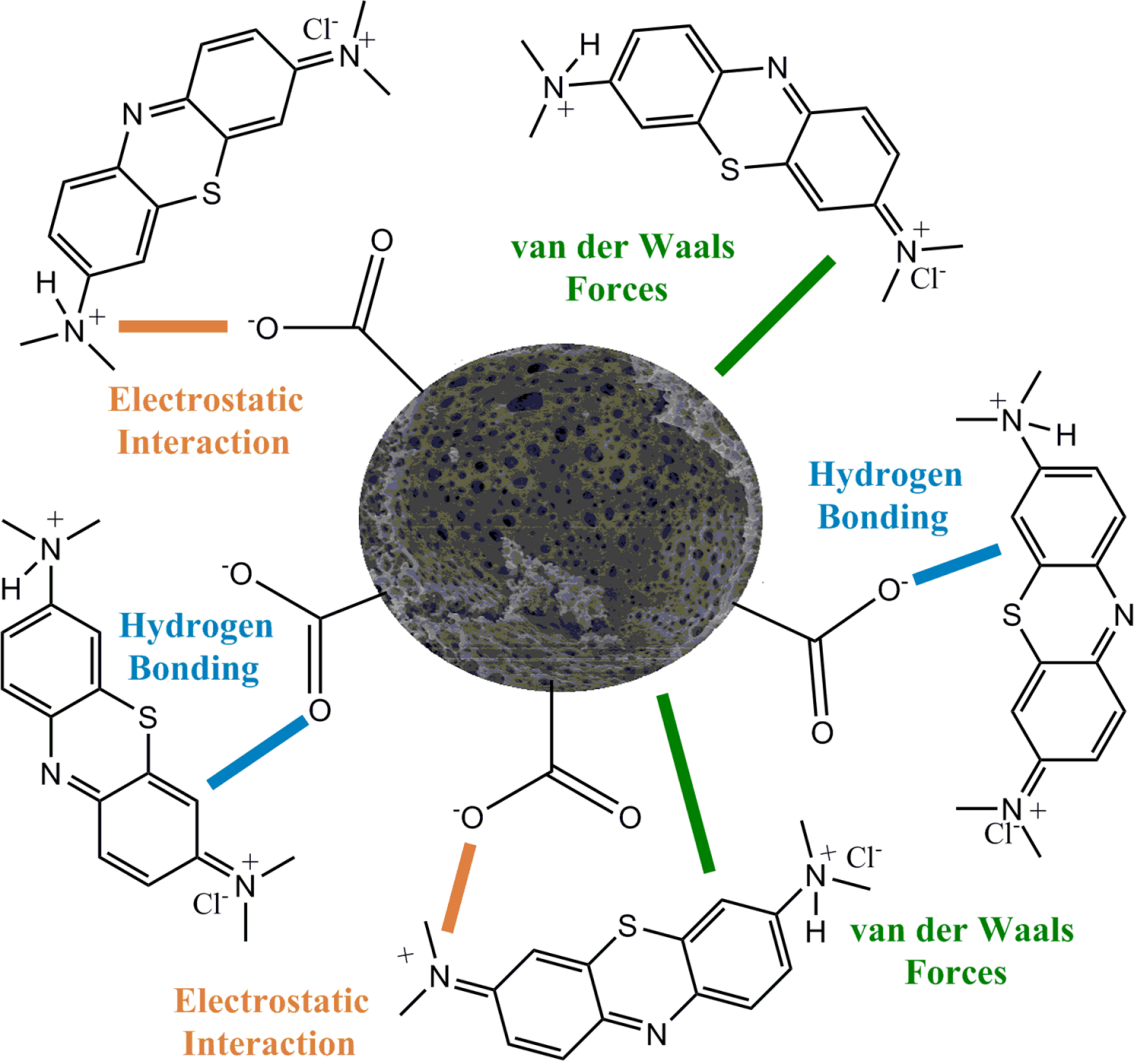
Probable interactions between the acidic functional polyHIPE and methylene blue.

Methylene blue is a basic dye. In aqueous solutions, the tertiary amine groups of dye molecules are positively charged by the protons released from the carboxylic acid groups of the acidic functional polyHIPE. The tertiary amine groups have a p*K*_a_ around 9–10, and the surface carboxylic acid groups have a p*K*_a_ of 4.76. At neutral pH, the polymer surface is negatively charged and the electrostatic forces between positively charged dye molecules and the negatively charged polymer surface occur. Through these electrostatic forces, the dye molecules can be retained on the polymer surface. Also, hydrogen bonds and van der Waals forces contribute to dye adsorption. In Figure 5, the probable interactions between the dye molecules and the porous polymer can be seen.^20,37^

First, a calibration curve for methylene blue at 663 nm was obtained in the concentration range between 1 and 10 ppm. As can be seen in Figure S1, the absorbance–concentration plot between 1 and 10 ppm shows linearity. A high correlation coefficient (*R*^2^ = 0.9971) was used in the methylene blue adsorption experiments in this range. The effect of surface area and pore structure on the methylene blue adsorption kinetics was studied for PHP-COOH, HXL-15 min-PHP-COOH, and HXL-60 min-PHP-COOH. All of the other experiments were carried out for HXL-15 min-PHP-COOH, which showed the best results.

#### 3.4.1 Effect of pH, Solid/Liquid Ratio (Determining the Optimal Adsorbent Amount) and Ionic Strength on Methylene Blue Adsorption

**Figure 6:**
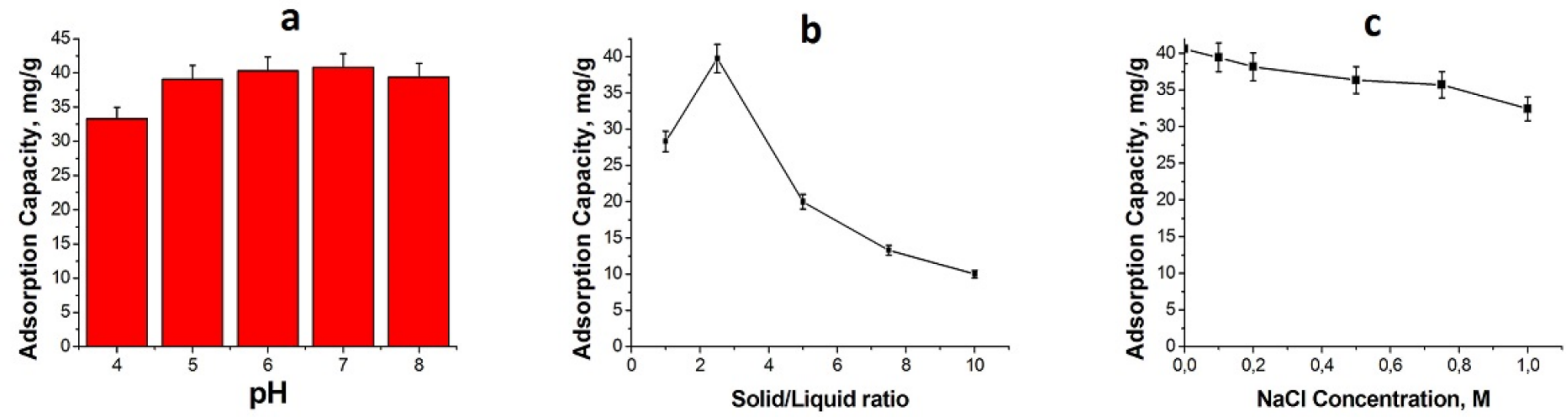
(a) Effect of pH, (b) solid/liquid ratio (solid: 10 to 100 mg of HXL-15 min-PHP-COOH and liquid: methylene blue solution), and (c) ionic strength on methylene blue adsorption. Adsorption conditions for pH: 25 mg of polymer, initial dye concentration = 100 ppm, volume of dye solution = 10 mL. Adsorption conditions for the solid/liquid ratio: initial dye concentration = 100 ppm, volume of dye solution = 10 mL. Adsorption conditions for ionic strength: 25 mg of polymer, initial dye concentration = 100 ppm, volume of dye solution = 10 mL.

The pH of the adsorption medium has a strong effect on dye adsorption. Depending on the pH of the adsorption medium, both the polymer surface and dye molecules are charged positively or negatively. The like or opposite charges effect the dye adsorption significantly on the polymer surface. The methylene blue adsorption on HXL-15 min-PHP-COOH was studied in a pH range of 4–8, and the adsorption capacities depending on pH of the adsorption medium are given in Figure 6. As can be seen from Figure 6a, HXL-15 min-PHP-COOH showed good adsorption capacities in a broad pH range. The best results came from pH 6 and 7, and the lowest adsorption capacity was observed at pH 4. These results were expected, and the experimental outcome met the theoretical expectations. At pH 6 and 7, the carboxylic acid groups on the polymer surface ionize and protonate the tertiary amine groups of methylene blue. The oppositely charged surface active groups and methylene blue interact, and the polymeric adsorbent shows a higher adsorption capacity. At pH 4, the polymer surface remains uncharged, and the electrostatic forces are not involved in the dye adsorption on the polymer, leading to a lower adsorption capacity. Although at pH 4 the electrostatic interactions do not contribute to the adsorption of methylene blue, due to the hydrogen bonding and van der Waals forces, the adsorbent still has an adsorption capacity greater than 30 mg/g at the 100 ppm initial dye concentration. As the pH of the adsorption medium increases, the positive charges of the dye molecules become weaker, resulting in a lower adsorption capacity at pH 8.

To identify an optimum amount for HXL-15 min-PHP-COOH, the effect of the solid/liquid ratio on methylene blue adsorption was studied. As can be seen in Figure 6b, the hyper-cross-linked polymer showed the highest dye adsorption capacity at a solid/liquid ratio of 2.5. As the solid/liquid ratio increases, the adsorption capacity decreases because the amount of dye molecules remains constant as the number of the active groups on the polymer surface increases. According to these results, for further adsorption experiments, 25 mg of adsorbent and 10 mL of dye solution were used. The main contribution for dye adsorption on the polymeric adsorbent comes from the electrostatic interactions. Therefore, the effect of ionic strength on methylene blue adsorption for HXL-15 min-PHP-COOH was investigated. As can be observed in Figure 6c, as the ionic strength (NaCl concentration) increases, the adsorption capacity of the polymeric adsorbent decreases. This result was expected due to the fact that the ionic strength of the adsorption medium weakens the ionic interactions between the oppositely charged polymer and dye. However, at a very high ionic strength (1 M NaCl), HXL-15 min-PHP-COOH lost its dye adsorption capacity by only 12.5%. These results indicate clearly that HXL-15 min-PHP-COOH can be used as an adsorbent for methylene blue in a broad range of pH values and ionic strength, which is important for real applications.

#### 3.4.2 Comparative Adsorption Kinetics

**Figure 7:**
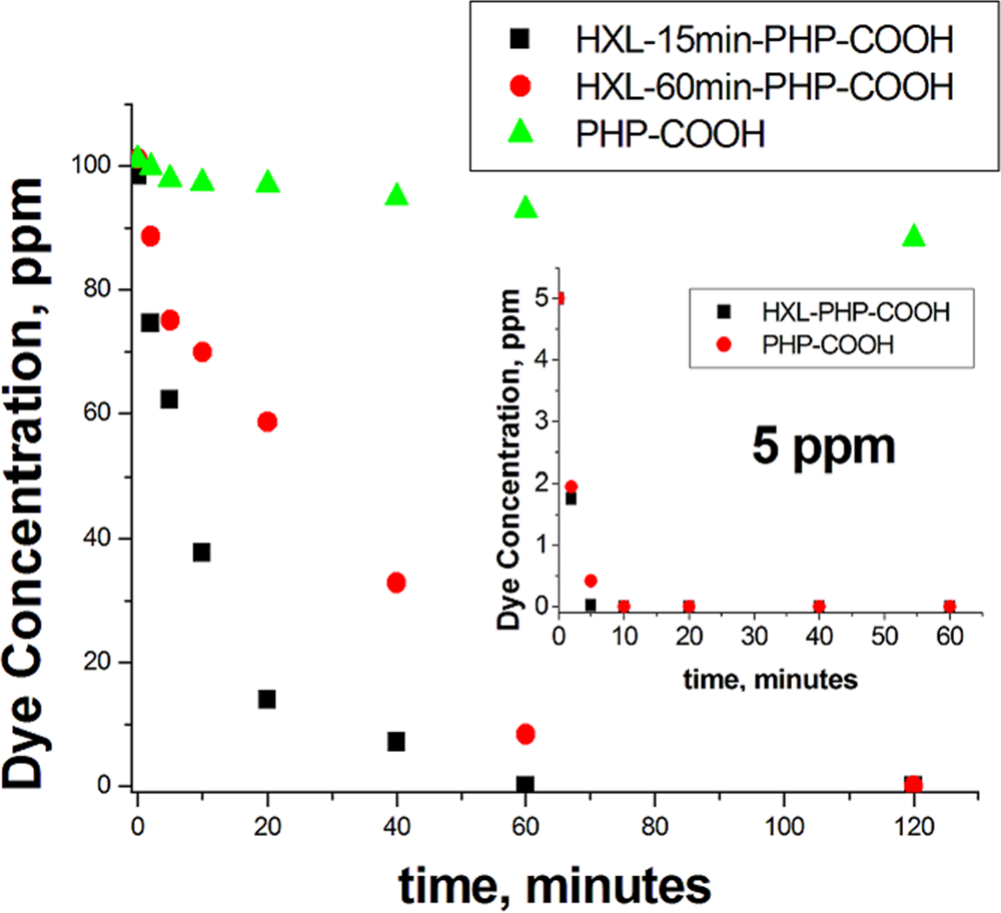
Comparative adsorption kinetics for methylene blue. Adsorption conditions: 25 mg of polymer, initial dye concentration = 100 ppm, volume of dye solution = 10 mL. Inset shows methylene blue adsorption comparison between HXL-15 min-PHP-COOH and PHP-COOH for 5 ppm initial dye concentration.

In this work, porous polymers having an interconnected network were prepared with high internal phase emulsion. The resulting polymer is a highly porous but a low-surface-area polymer. Therefore, the surface area of the resulting polymer was increased, and the adsorption performances of the various polymers were compared kinetically. Because the hyper-cross-linking reaction is a controlled reaction, two hyper-cross-linked polyHIPEs were prepared for 15 and 60 min reaction times, and their adsorption performances were compared with those of the unhyper-cross-linked counterparts (Figure 7). First, the adsorption kinetics of PHP-COOH and HXL-15 min-PHP-COOH were compared using an initial concentration of 5 ppm methylene blue. In this first attempt, both polymeric adsorbents showed very fast kinetics. Both polymers lowered an initial 5 ppm dye concentration to 0 in just 10 min. Although this result clearly proves that carboxylic acid functional polyHIPEs are good candidates for methylene blue adsorption, to show the advantage of the hyper-cross-linked polyHIPE and to evaluate the adsorption kinetics mechanism, the adsorption experiments were performed at higher concentrations. Moreover, it was possible to obtain an optimal hyper-cross-link degree by comparative adsorption kinetics experiments. As can be seen in Figure 7, the 15 min hyper-cross-linking reaction enhanced the methylene blue adsorption significantly. When the comparative adsorption experiments were performed at an initial methylene blue concentration of 100 ppm, the adsorption performances between the hyper-cross-linked and unhyper-cross-linked polymers became very clear. Whereas HXL-15 min-PHP-COOH was able to decrease the total initial dye concentration (100 ppm) to 0 in 60 min, its unhyper-cross-linked counterpart could decrease the same initial dye concentration by just 10%. It can be concluded from this result that the hyper-cross-linking reaction can enhance the adsorption performance of polyHIPE, and HXL-15 min-PHP-COOH is an effective adsorbent even at high concentrations. Another comparison was made between the hyper-cross-linked polymers prepared for 15 and 60 min hyper-cross-linking reactions. As can be seen in Figure 7, HXL-60 min-PHP-COOH adsorbed methylene blue completely in 120 min. Although the adsorption performance of HXL-60 min-PHP-COOH is much better than that of PHP-COOH, its adsorption rate was found to be slower than that of HXL-15 min-PHP-COOH. The hyper-cross-linked polymers can adsorb dye molecules through the active groups located in their hierarchical pore structure composed of voids, windows, mesopores, and micropores. The general expectation is that the higher the surface area of an adsorbent, the faster the adsorption. However, for a fast adsorption rate, all active groups in the pores need to be accessible to adsorbates. As a result of hyper-cross-linking, very narrow micropores may not be accessible to dye molecules. As the degree of hyper-cross-linking increases the amount of micropores, inaccessible active groups increase, resulting in a slower adsorption rate. Therefore, an optimal hyper-cross-linking degree needs to be found for better adsorption performance. According to the comparative adsorption results, the hyper-cross-linking reaction for 15 min was found to be the optimum.

#### 3.4.3 Evaluating Dye Adsorption Mechanism

To evaluate the adsorption mechanism, three adsorption kinetics models were applied to the experimental adsorption kinetics data at various initial dye concentrations. These models are pseudo-first-order, pseudo-second-order, and intraparticle diffusion models. Because these models can describe the adsorption kinetics in a limited concentration range, the kinetics experiments were carried out at various initial concentrations. To apply the models, first, adsorption capacities for HXL-15 min-PHP-COOH at various time intervals were calculated (Figure S2).

**Figure 8:**
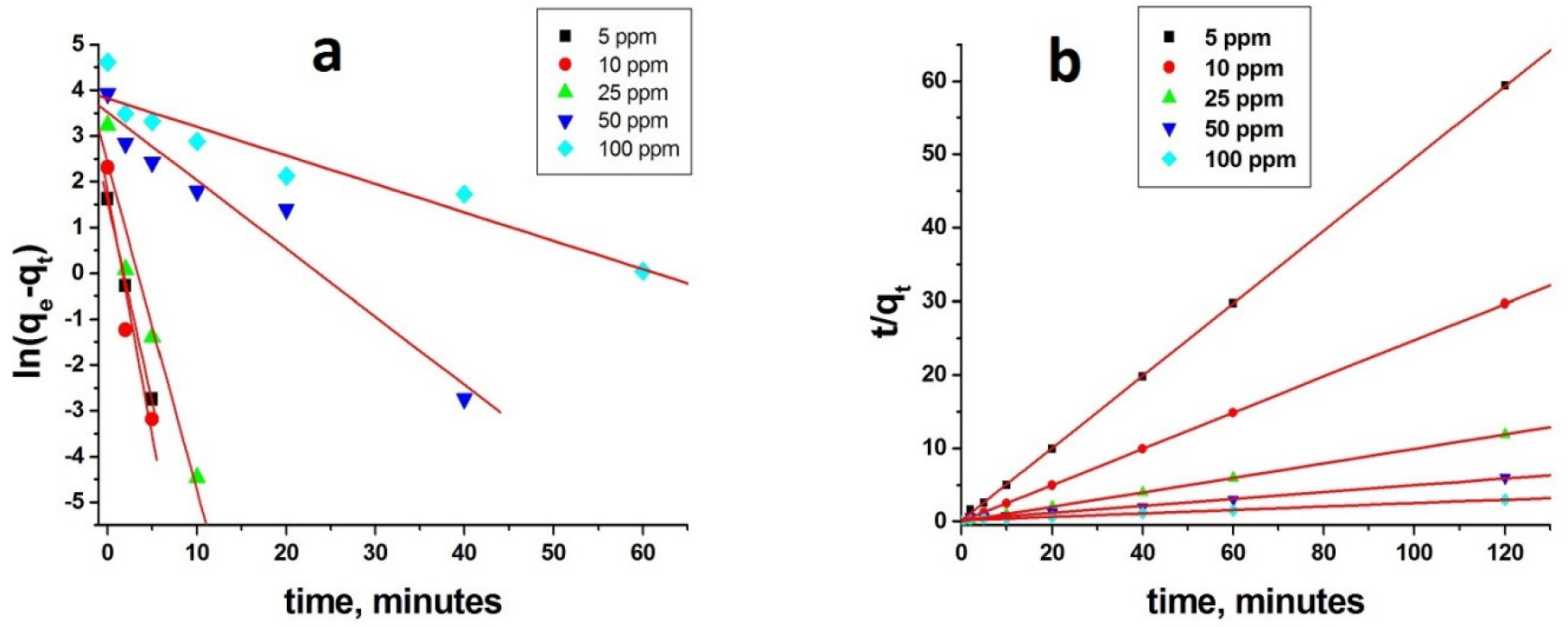
(a) Pseudo-first-order kinetics plot and (b) pseudo-second-order kinetics plot for methylene blue adsorption on HXL-15 min-PHP-COOH. Adsorption conditions: 25 mg of polymer, initial dye concentration = 100 ppm, volume of dye solution = 10 mL.

The Lagergren first-order rate equation is a well-known equation for the adsorption process.^38^ The linear form of the equation is given as follows:
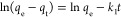
1Here, in this equation, *k*_1_ is the pseudo-first-order rate constant for adsorption, and *q*_eq_ and *q*_t_ indicate the amounts of adsorption (mmol g^–1^) at equilibrium and at time *t*, respectively. The rate constant and *q*_e_ were calculated from the slope and intercept of the ln(*q*_eq_ – *q*_t_)–time plot, respectively. The second kinetic model used in this study is the pseudo-second-order model.^39^ The linear form of the equation describing the adsorption kinetics is as follows:
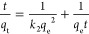
2Here, *k*_2_ is the pseudo-second-order rate constant (g mmol^–1^ min^–1^); *k*_2_ and the equilibrium capacity *q*_e_ can be calculated from the intercept and slope of the *t*/*q*_t_–time plot, respectively. The plot showed linearity with a high correlation factor, which allowed *k*_2_ and *q*_e_ to be achieved. The pseudo-first-order and pseudo-second-order kinetics plots and linear regressions for the methylene blue adsorption on HXL-15 min-PHP-COOH are given in Figure 8a,b, respectively.

**Figure 9:**
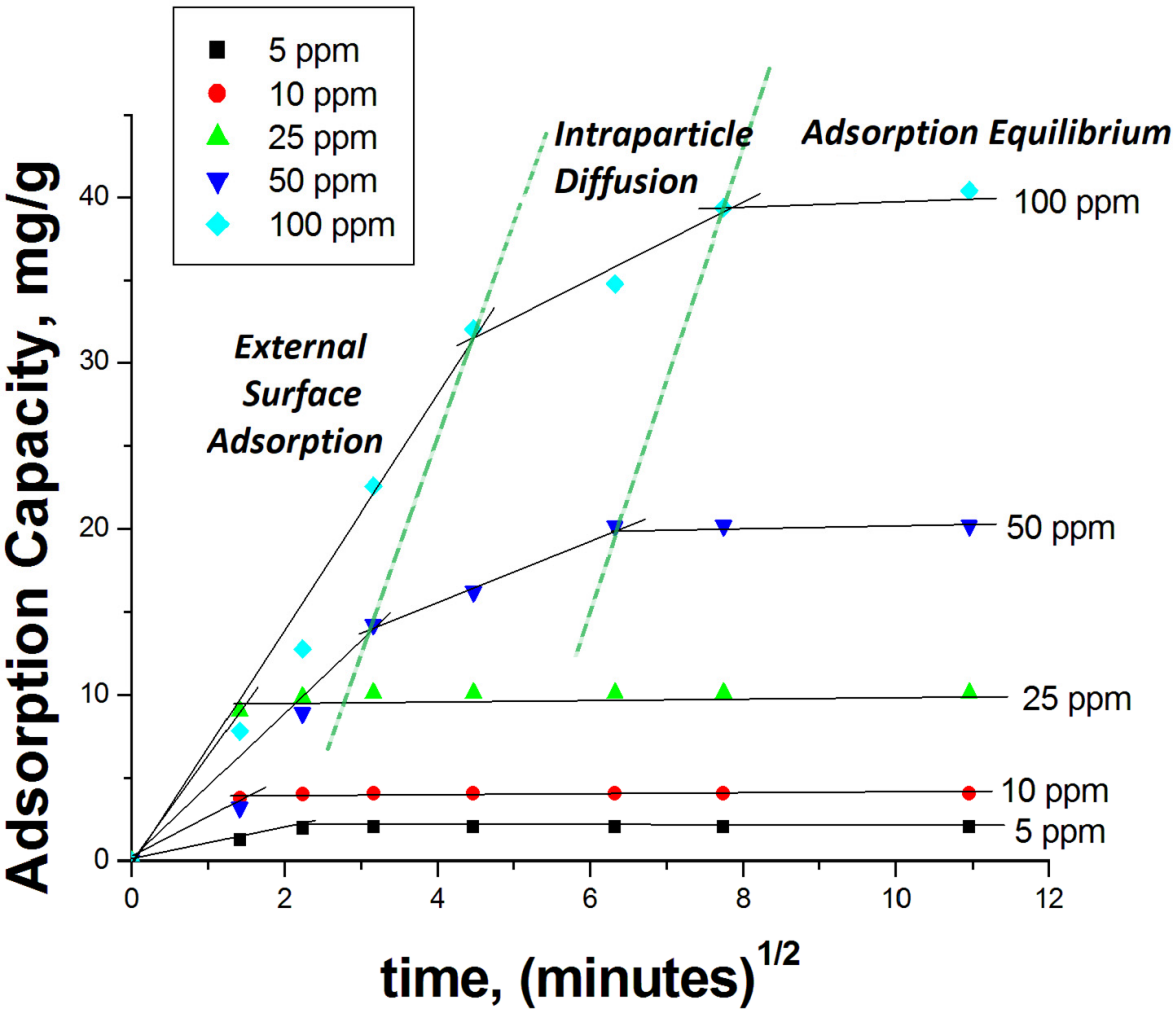
Intraparticle diffusion plots for the methylene blue adsorption on HXL-15 min-PHP-COOH. Adsorption conditions: 25 mg of polymer, initial dye concentration = 100 ppm, volume of dye solution = 10 mL.

It can be evaluated which model describes the methylene blue adsorption on HXL-15 min-PHP-COOH judging by the correlation coefficients of the corresponding plots. In Table 3, we can observe the kinetics parameters calculated by linear regression. As can be seen in Table 3, methylene blue adsorption obeys the second-order kinetics for all initial dye concentrations. The high correlation coefficients and the close experimental and theoretical adsorption capacity values indicate that the adsorption process can be explained by the pseudo-second-order kinetics model. However, we can observe from the table that the experimental data fit well with the pseudo-first-order kinetic model at low dye concentrations. For an initial dye concentration of 5 ppm, a high correlation coefficient was obtained. Moreover, in this case, the experimental and theoretical *q* values are close to each other. The adsorption kinetics at initial dye concentrations higher than 5 ppm are not in good agreement with the pseudo-first-order kinetic model. The pseudo-second-order kinetic model describes the adsorptions where the adsorbate/adsorbent ratio is low and the rate-limiting step is chemical adsorption. Due to the fact that methylene blue is adsorbed on the polymer surface through mainly electrostatic interactions, the second-order kinetics model is preferential for our adsorption process. As can be seen in Table 3, for low dye concentrations such as 5, 10, and 25 ppm, very high correlation coefficients and very small differences between the experimental and theoretical adsorption capacities were observed. However, as the adsorbate/adsorbent ratio increases (50 and 100 ppm), *k*_2_ values show high deviations from the values calculated for the first three concentrations. The second-order rate constants obtained from the adsorption experiments with 5, 10, and 25 ppm are very close to each other. Because the reaction rate constant is independent of adsorbate concentration, the second-order model can be applied to methylene blue adsorption on HXL-15 min-PHP-COOH at the concentrations of 5, 10, and 25 ppm. The third kinetic model used to describe the adsorption process in this study is the intraparticle diffusion (Figure 9).

**Table 3:** Kinetics Parameters for Methylene Blue Adsorption on HXL-15 min-PHP-COOH

	First Order				Second Order		
	*q*_eq_,exp (mg g^–1^)	*k*_1_ × 10^2^ (min^–1^)	*q*_e_ (mg g^–1^)	*R*^2^	*k*_2_ (g mg^–1^ min^–1^)	*q*_e_ (mg g^–1^)	*R*^2^
5 ppm	2.0	87.0	2.75	0.9985	1.7400	2.03	0.9999
10 ppm	4.0	100.6	5.93	0.9535	1.8500	4.05	1
25 ppm	10.1	71.5	11.1	0.9477	1.6200	10.1	1
50 ppm	20.2	14.9	33.5	0.9511	0.0088	21.3	0.9930
100 ppm	40.4	6.20	45.5	0.9093	0.0031	43.5	0.9928

The adsorption medium containing the porous adsorbent and dye solution should be stirred well to apply this model to the experimental data. The intraparticle diffusion model was proposed by Weber and Morris,^40^ and it is given as following equation:
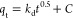
3Here, *k*_d_ represents the intraparticle diffusion rate constant (g mmol^–1^min^–0.5^), and *C* is the constant of the boundary layer thickness. The multilinear *q*_t_–*t*^0.5^ plots are generally composed of two or three steps. In the first step, a sudden increase in the adsorption capacity takes place, and this portion of the plot is called the external surface adsorption. In the second part of the plot, a slow adsorption stage takes place, where intraparticle diffusion is rate-controlled. In the final portion, because of very low concentrations in the solution, the intraparticle diffusion begins to slow down and the equilibrium stage is established. As can be seen in Figure 9, the plots for 5, 10, and 25 ppm show two stages. In these cases, a very fast external surface adsorption takes place, followed by the equilibrium stage. Although we observed all three stages for 50 and 100 ppm initial dye concentrations, the intraparticle diffusion stage was completed very fast. These results indicate that the active groups located in the open-porous structure of the hyper-cross-linked polyHIPE are accessible for dye molecules and the resistance to mass transfer as pore diffusion is low. For 5, 10, and 25 ppm initial dye concentrations, this resistance completely disappeared, and for 50 and 100 ppm, it was very low. To find the maximum adsorption capacity for HXL-15 min-PHP-COOH, the adsorption experiments with various initial methylene blue concentrations were carried out. These results were tested by three adsorption isotherm models. As can be seen in Figure S3, as the dye concentration increases, the adsorption capacity of HXL-15 min-PHP-COOH increases and reaches a maximum level at 1500 ppm dye concentration, indicating a 450 mg/g capacity.

The first adsorption model applied to the experimental adsorption data in this study is the Langmuir isotherm model, which may be represented as
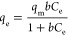
4To evaluate the experimental data with the Langmuir isotherm, the linear form of the isotherm was used:
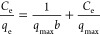
5Here, *C*_e_ shows the equilibrium concentration of dye in solution (mg L^–1^), and *q*_e_ indicates the equilibrium amount of dye adsorbed on the adsorbent at time *t* (mg g^–1^); *q*_m_ is the maximum adsorption capacity of the adsorbent (mg g^–1^), and *b* is the reciprocal of dissociation constant *K*_d_. When *K*_d_ is small, the dye binding is stronger. In Figure S4, the Langmuir isotherm of methylene blue adsorption on HXL-15 min-PHP-COOH is given. The second adsorption isotherm used in this study is the Freundlich isotherm, which is applied for the heterogeneous surface adsorption. The linear form of the Freundlich equation is represented as
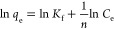
6In this equation, *K*_F_ and *n* are the Freundlich constants, and they are indicators of the characteristic of the adsorption system. *K*_F_ and *n* indicate the adsorption capacity and adsorption intensity, respectively. The slope of the linear equation is 1/*n*, and the intercept of the equation gives ln *K*_F_. Figure S5 represents the Freundlich isotherm plotted using the methylene blue adsorption data for HXL-15 min-PHP-COOH. The experimental adsorption data were applied to the D–R as the third adsorption isotherm model. Generally, polymeric adsorbents have a heterogeneous surface, and the active groups are not distributed regularly on the polymer surface. Because the D–R isotherm does not assume a homogeneous surface or constant adsorption potential,^41^ we also used this isotherm to evaluate the adsorption mechanism of methylene blue on HXL-15 min-PHP-COOH. The following equation is the D–R equation:
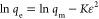
7In this equation, *q*_e_ indicates the amount of the dye adsorbed at the equilibrium; *K* is a constant which is related to the mean free energy of sorption; *q*_m_ gives the theoretical adsorption capacity, and ε is the Polanyi potential. The value of ε can be calculated with the equation: *RT* ln(1 + (1/*C*_e_)). The plot of ln *q*_e_ against ε^2^ gives the values of *q*_m_ and *K*. The constant (*K*) is an indicator of the mean free energy of adsorption per mole of the adsorbate. The following equation can be used to calculate the mean free energy (*E*).
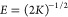
8In Figure S6, the D–R isotherm for methylene blue adsorption on HXL-15 min-PHP-COOH was given. The isotherm parameters and correlation coefficients for HXL-15 min-PHP-COOH are given in Table S1. According to the correlation factors, the experimental data are fitted well with both Langmuir and Freundlich isotherms. For both linear isotherm models, the high correlation factors were obtained. Moreover, *q*_max_ calculated from the Langmuir model is close to the experimental maximum dye adsorption capacity. Because of a significant deviation from linearity for the D–R model, *q*_m_ and *E* values were not calculated.

**Figure 10:**
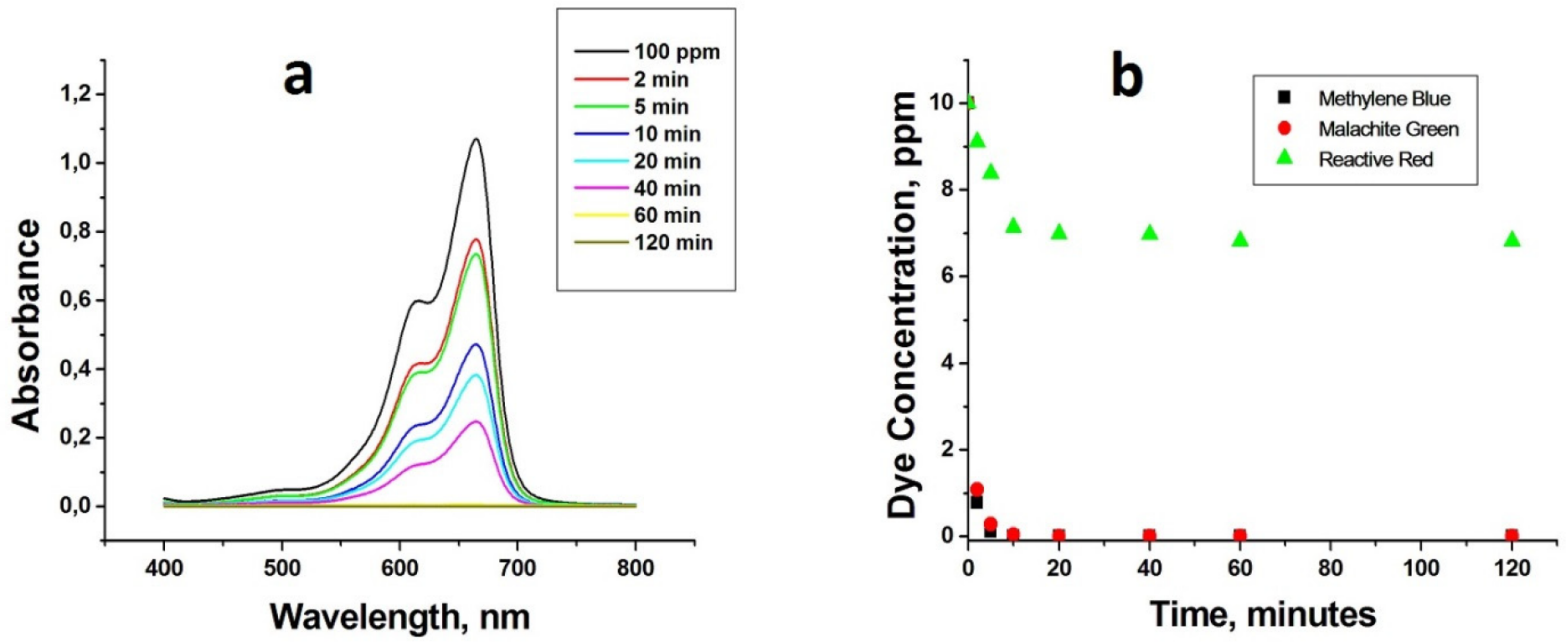
(a) UV–vis spectra of the methylene blue adsorption on HXL-15 min-PHP-COOH. Adsorption conditions: 25 mg of polymer, initial dye concentration = 100 ppm, volume of dye solution = 10 mL, pH 7. (b) Selectivity of dye adsorption on HXL-15 min-PHP-COOH. Adsorption conditions: 25 mg of polymer, initial dye concentration = 10 ppm, volume of dye solution = 10 mL, pH 7.

To evaluate the affinity of the carboxylic acid functional hyper-cross-linked polyHIPE toward methylene blue, the adsorption of malachite green and reactive red dyes was determined with a UV–vis spectrophotometer. As can be seen in Figure 10a and Figure S7, methylene blue concentration (50 ppm in Figure S7 and 100 ppm in Figure 10) decreases regularly with time and falls to 0 in 120 min. HXL-15 min-PHP-COOH shows high adsorption kinetics for malachite green; however, its adsorption performance was found to be poor in the case of reactive red (Figure 10b). For an initial dye concentration of 10 ppm, as the adsorbent could be able to adsorb methylene blue and malachite green completely in just 10 min, the reactive red concentration dropped to around 7 ppm in 120 min. When the pH of the adsorption medium is 7, basic dyes methylene blue and malachite green are positively charged, and the polymer surface is negatively charged. On the other hand, acidic dye reactive red is negatively charged at pH 7. Therefore, the adsorbent did show a poor adsorption performance for reactive red as a result of the repulsive forces between the negatively charged polymer surface and dye molecules.

#### 3.4.4 Regeneration of the PolyHIPE Adsorbent

**Figure 11:**
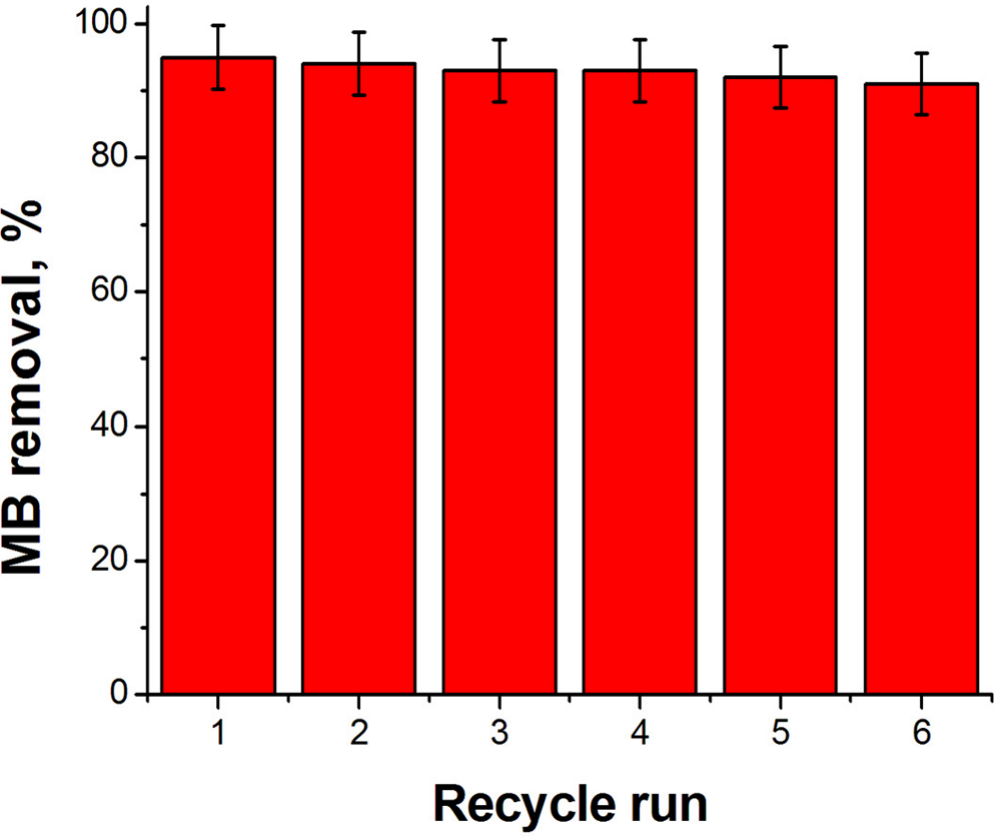
Reusability of HXL-15 min-PHP-COOH.

To test the recyclability of the porous adsorbent, HXL-15 min-PHP-COOH, it was isolated from the adsorption solution through filtration under gravity followed by washing it with water to remove unbound dyes. Then the dye-loaded polymer was charged with a HCl (1 M)/ethanol solution (10 mL, 50%, v/v) to regenerate the active sites. After desorption, the adsorbent was washed with excess water and reused for another cycle. The adsorbent was used for MB adsorption at least six times without losing its adsorption capacity significantly by following this procedure (Figure 11).

## 4 Conclusion

In this work, to obtain an acidic functional, highly porous adsorbent, high internal phase emulsions (HIPEs) were used as an emulsion templated method. This emulsion system was polymerized, and the resulting open-porous material (polyHIPE) was used as an adsorbent for methylene blue adsorption. To characterize the material, SEM, XPS, FTIR, BET, and analytical methods were successfully used. The cross-linked highly porous adsorbent showed a high adsorption capacity and fast uptake for the cationic dye, methylene blue. The comparative adsorption kinetics for methylene blue were studied using the variants of these polymers by cross-linking. These polymers were represented by PHP-COOH for the unhyper-cross-linked acidic functional polymer, HXL-15 min-PHP-COOH for the polymer prepared with a 15 min hyper-cross-linking reaction, and HXL-60 min-PHP-COOH for the polymer prepared with a 60 min hyper-cross-linking reaction. The hyper-cross-linking method allowed the surface area to be increased, and the optimal hyper-cross-linking was determined through the comparative dye adsorption experiments. It was found that HXL-15 min-PHP-COOH showed better adsorption performance for methylene blue. The effects of pH, ionic strength, initial dye concentration, and solid/liquid ratio on dye adsorption for HXL-15 min-PHP-COOH were studied. The hyper-cross-linked polyHIPE can also be reused after desorption with acid treatment, which is an advantage of these materials. The maximum adsorption capacity of the adsorbent was found to be 450 mg/g for methylene blue. As a result of these observations, it was concluded that the hyper-cross-linked, acidic functional polyHIPE adsorbent is a very good candidate for use as an adsorbent for methylene blue.

## Supporting Information Available

Calibration curve for methylene blue; adsorption capacity versus time plot and adsorption capacity–dye concentration plot for HXL-15 min-PHP-COOH; isotherm plots for the methylene blue adsorption on HXL-15 min-PHP-COOH; table of the isotherm parameters and correlation coefficients; UV–vis spectra of methylene blue adsorption on HXL-15 min-PHP-COOH (PDF)
