# Supplementary material for: Emulsion-Templated Porous Polymers for Efficient Dye Removal
Source: ACS Omega. 2022 Apr 29;7(18):16127–40. doi: 10.1021/acsomega.2c01472 (PMC9097204; doi:10.1021/acsomega.2c01472)
Supplement: Supplementary file 1 — ao2c01472_si_001.pdf [file ao2c01472_si_001.pdf]

Supporting Information

Emulsion-Templated Porous Polymers for Efficient Dye Removal

*Gülenay Üzüm, Büşra Akın Özmen, Ebru Tekneci Akgül, Erdem Yavuz**

[1]Department of Chemistry, Istanbul Technical University, Maslak-Istanbul, 34469, Turkey

*Email: yavuzerd@itu.edu.tr

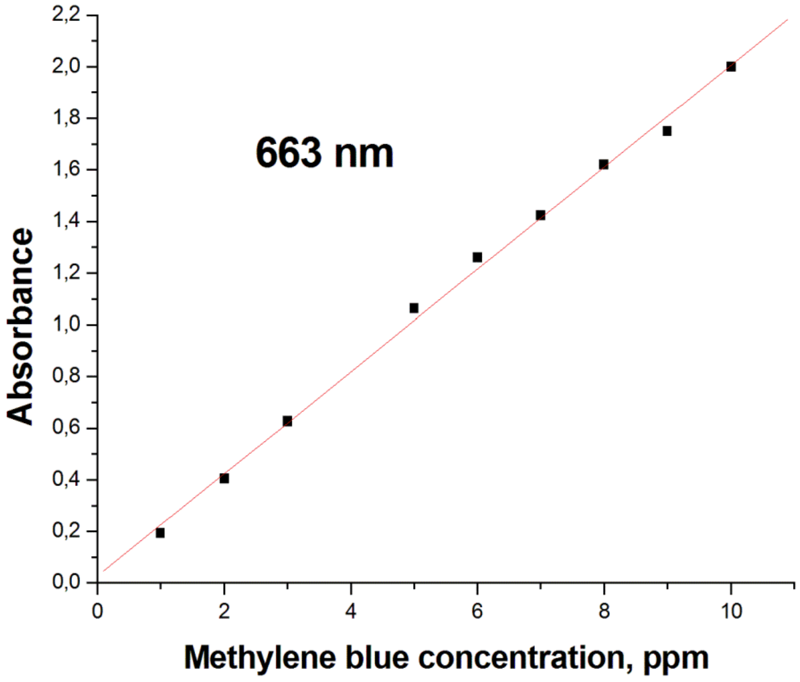

Figure S1. The calibration curve for methylene blue

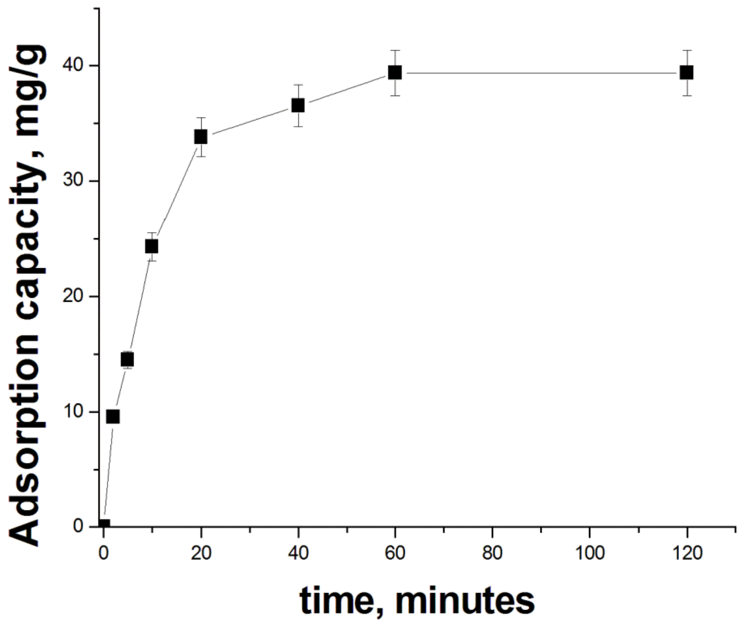

Figure S2. Adsorption capacity versus time plot for HXL-15min-PHP-COOH. Adsorption conditions: 25 mg polymer, the initial dye concentration = 100 ppm, the volume of dye solution = 10 mL.

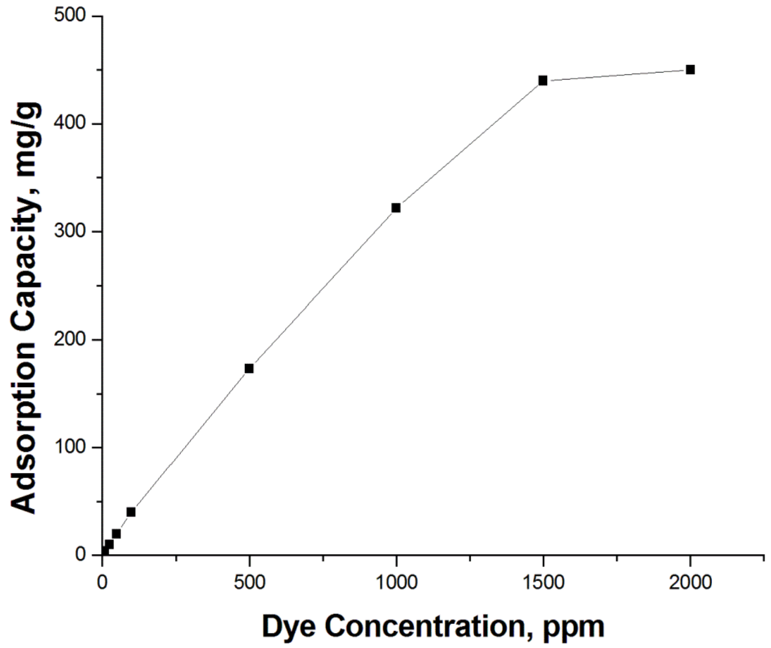

Figure S3. Adsorption capacity – dye concentration plot for HXL-15min-PHP-COOH. Adsorption conditions: 25 mg polymer, the volume of dye solution = 10 mL.

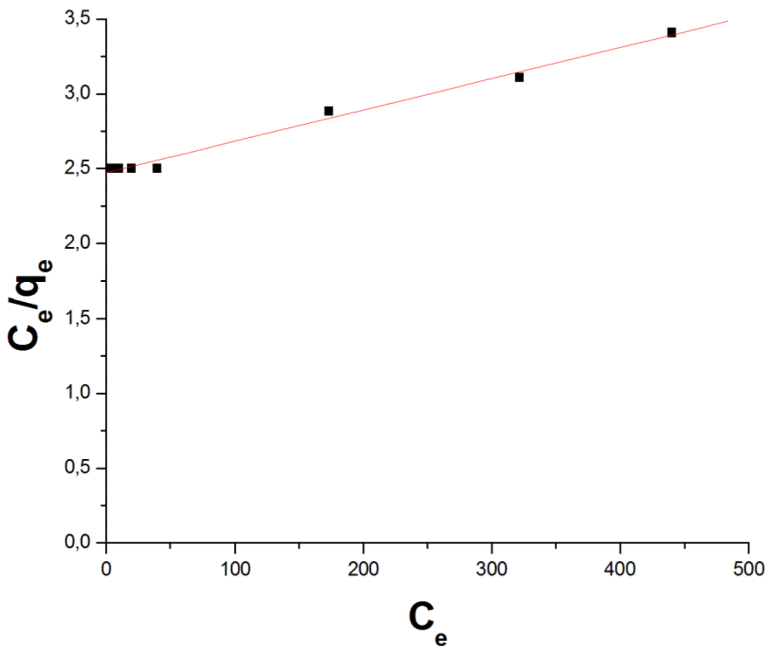

Figure S4. The langmuir isotherm for methylene blue adsorption on HXL-15min-PHP-COOH.

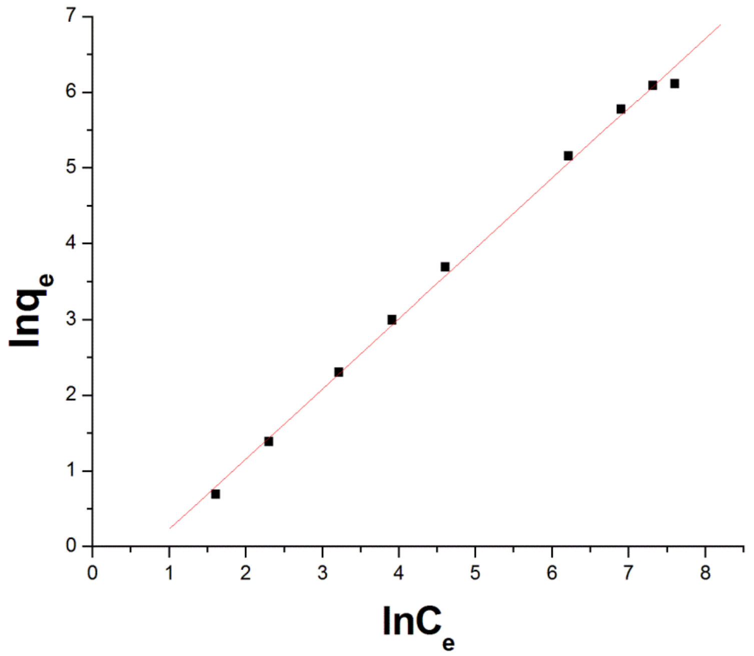

Figure S5. The Freundlich isotherm for methylene blue adsorption on HXL-15min-PHP-COOH.

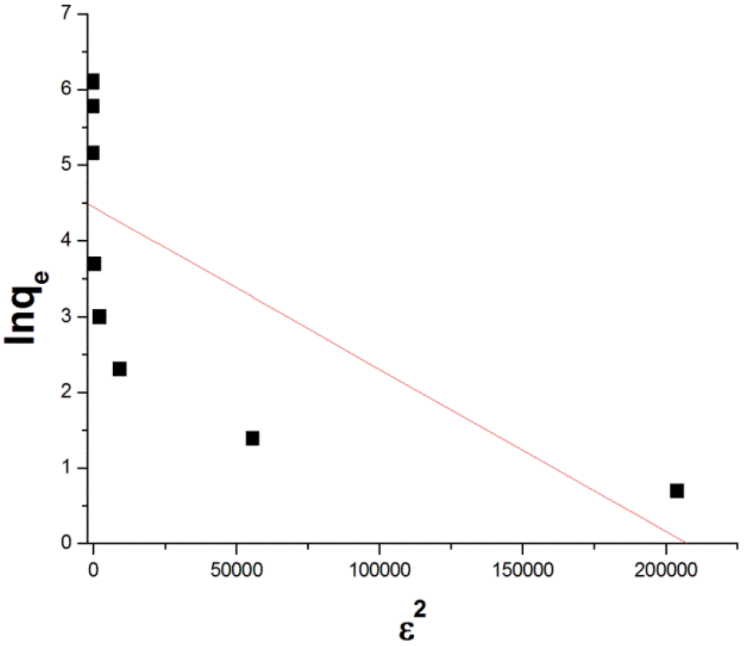

Figure S6. The D-R isotherm for methylene blue adsorption on HXL-15min-PHP-COOH

Table S1.The isotherm parameters and correlation coefficients for methylene blue adsorption on HXL-15min-PHP-COOH.

	Langmuir			Freundlich			D-R		
q_{exp} (mg g^{-1})	K_d (mg L^{-1})	q_m (mg g^{-1})	R^2	K_f (L g^{-1})	n	R^2	q_m (mg g^{-1})	E (kcal mol^{-1})	R^2
450	1087	478.5	0.9906	0.219	1.08	0.9971	-	-	0.4847

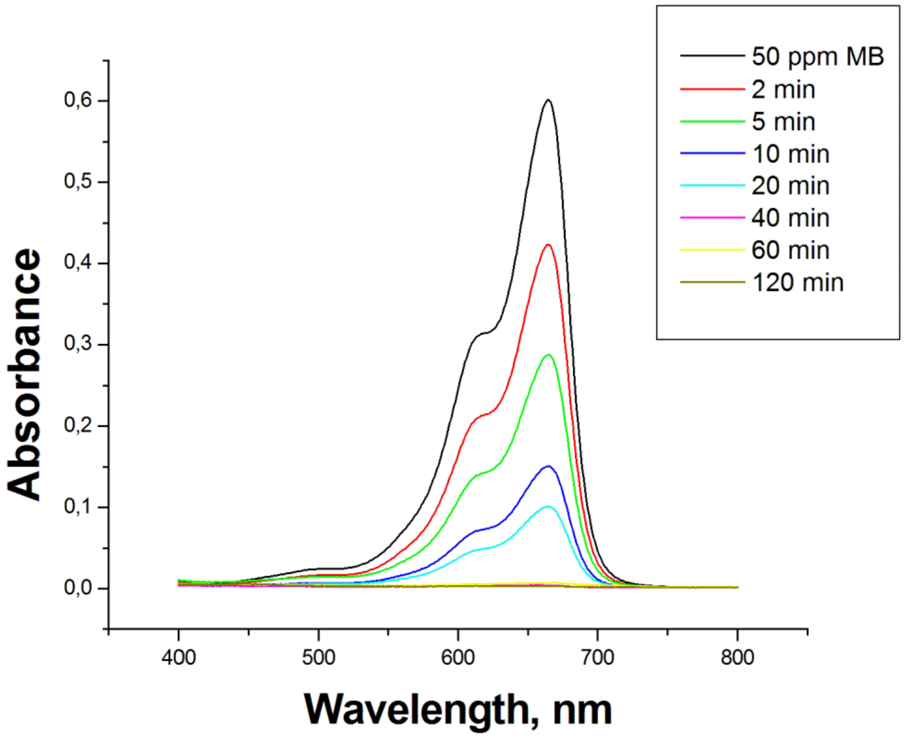

Figure S7. UV-Vis spectrums of methylene blue adsorption on HXL-15min-PHP-COOH. Adsorption conditions: 25 mg polymer, the initial dye concentration = 50 ppm, the volume of dye solution = 10 mL, pH = 7.
